# Short-term genome evolution of *Listeria monocytogenes *in a non-controlled environment

**DOI:** 10.1186/1471-2164-9-539

**Published:** 2008-11-13

**Authors:** Renato H Orsi, Mark L Borowsky, Peter Lauer, Sarah K Young, Chad Nusbaum, James E Galagan, Bruce W Birren, Reid A Ivy, Qi Sun, Lewis M Graves, Bala Swaminathan, Martin Wiedmann

**Affiliations:** 1 Department of Food Science, Cornell University, Ithaca, USA; 2 Genome Sequencing and Analysis Program, Broad Institute of MIT and Harvard, Cambridge, USA; 3 Anza Therapeutics, Concord, USA; 4 Department of Biomedical Engineering and Microbiology, Boston University, USA; 5 Computational Biology Services Unit, Center for Advanced Computing, Cornell University, Ithaca, USA; 6 Enteric Diseases Laboratory Branch, Division of Foodborne, Bacterial and Mycotic Diseases, Centers for Disease Control and Prevention, Atlanta, USA; 7 Department of Molecular Biology, Massachusetts General Hospital, Boston, USA

## Abstract

**Background:**

While increasing data on bacterial evolution in controlled environments are available, our understanding of bacterial genome evolution in natural environments is limited. We thus performed full genome analyses on four *Listeria monocytogenes*, including human and food isolates from both a 1988 case of sporadic listeriosis and a 2000 listeriosis outbreak, which had been linked to contaminated food from a single processing facility. All four isolates had been shown to have identical subtypes, suggesting that a specific *L. monocytogenes *strain persisted in this processing plant over at least 12 years. While a genome sequence for the 1988 food isolate has been reported, we sequenced the genomes of the 1988 human isolate as well as a human and a food isolate from the 2000 outbreak to allow for comparative genome analyses.

**Results:**

The two *L. monocytogenes *isolates from 1988 and the two isolates from 2000 had highly similar genome backbone sequences with very few single nucleotide (nt) polymorphisms (1 – 8 SNPs/isolate; confirmed by re-sequencing). While no genome rearrangements were identified in the backbone genome of the four isolates, a 42 kb prophage inserted in the chromosomal *comK *gene showed evidence for major genome rearrangements. The human-food isolate pair from each 1988 and 2000 had identical prophage sequence; however, there were significant differences in the prophage sequences between the 1988 and 2000 isolates. Diversification of this prophage appears to have been caused by multiple homologous recombination events or possibly prophage replacement. In addition, only the 2000 human isolate contained a plasmid, suggesting plasmid loss or acquisition events. Surprisingly, besides the polymorphisms found in the *comK *prophage, a single SNP in the tRNA Thr-4 prophage represents the only SNP that differentiates the 1988 isolates from the 2000 isolates.

**Conclusion:**

Our data support the hypothesis that the 2000 human listeriosis outbreak was caused by a *L. monocytogenes *strain that persisted in a food processing facility over 12 years and show that genome sequencing is a valuable and feasible tool for retrospective epidemiological analyses. Short-term evolution of *L. monocytogenes *in non-controlled environments appears to involve limited diversification beyond plasmid gain or loss and prophage diversification, highlighting the importance of phages in bacterial evolution.

## Background

Experimentally evolved lineages of bacteria have provided important data on mechanisms involved in short-term bacterial evolution and adaptation [1-3] in simple controlled environments. However, experiments in controlled environments cannot represent all forces contributing to evolution in non-controlled settings and natural environments. Important differences between experimental and natural bacterial populations include (i) population sizes that are much greater in experimental populations than in a natural population; (ii) generation times that are probably much shorter in an experimental population than in a natural population; and (iii) absence of donors of genetic materials, which allow for rapid diversification via horizontal gene transfer (e.g., other bacteria, bacteriophages). In addition, while experimental populations experience selective pressures that are usually low in number but high in intensity, natural populations encounter many selective pressures imposed by physical (e.g., temperature), chemical (e.g., pH), and biological (e.g., phages and other microbial organisms) variables, which differ in intensity and vary over time. The strong selective pressure provided by controlled environments often results in parallel evolution of replicates under the same conditions [1,4,5]. For example, 12 *Escherichia coli *populations that originated from the same parent strain all showed substitutions in the same candidate genes after they were cultured for 20,000 generations under identical conditions [5].

*Listeria monocytogenes *is a gram-positive, facultative intracellular foodborne pathogen that causes listeriosis in humans and many mammalian and avian species [6,7]. Human listeriosis is a rare but severe disease; an estimated 2,500 human invasive listeriosis cases including 500 deaths occur annually in the United States [8]. The vast majority of human listeriosis cases are caused by foodborne transmission [8]. A common source of food contamination appears to be transmission of *L. monocytogenes *present in processing plant environments to food products after they have been heat processed [9]. *L. monocytogenes *is ubiquitously distributed in the environment. It can grow under a wide range of environmental conditions, including temperatures ranging from 0.4°C to 45°C [10,11] and pH ranging from 4 to 9.6 [12,13]. Consequently, it is difficult to control *L. monocytogenes *in food processing environments. Although food processing facilities and equipment are regularly cleaned and sanitized, persistence of specific *L. monocytogenes *subtypes, over time periods ranging from a few months to more than five years, has been documented for a number of food processing facilities [14-17].

It is also common for several *L. monocytogenes *subtypes to co-exist in the same food processing facility [15-20]. While use of molecular subtyping methods, such as pulsed-field gel electrophoresis (PFGE), has been critical in listeriosis outbreak investigations and surveillance, interpretation of subtyping data sometimes remains a challenge as isolates may diversify rapidly (e.g., due to plasmid losses, yielding different PFGE patterns [21]) and as epidemiologically unrelated strains may share identical PFGE patterns [22,23]. Consequently, there is a need to better understand *L. monocytogenes *diversification in non-controlled environments and to develop improved subtyping approaches that can be used in follow-up studies to routine subtyping using PFGE.

In 1988, a sporadic case of human listeriosis in Oklahoma, USA was linked to the consumption of *L. monocytogenes *contaminated turkey franks produced in a food processing facility in Texas, USA [24]. Twelve years later, in 2000, a multi-state listeriosis outbreak, which caused illnesses in 29 persons in 11 US states (including 4 deaths), was linked to consumption of deli turkey meat produced in the same processing facility that was linked to the 1988 listeriosis case [25,26]. Phenotypic and genotypic typing showed that human and food isolates from both episodes belonged to the same serotype (1/2a), were slow rhamnose fermenters (a rare phenotype for 1/2a isolates), and were indistinguishable by ribotyping (all isolates were ribotype DUP-1053A [27]) and PFGE [26]. The PFGE type for these isolates (type 25, as reported by [27]) was only found once among a set of 495 *L. monocytogenes *isolates previously characterized by PFGE [22], indicating that this specific strain represents a combination of genetic and phenotypic characteristics that is rare. While re-introduction of this strain in the processing plant from the outside environment represents a possibility, the rarity of this strain and the fact that ready-to-eat food processing plants typically implement practices to prevent introduction of microbial organisms suggests that persistence of a single *L. monocytogenes *strain in this facility for at least 12 years [14] is the most likely explanation for the observations detailed above. The genome sequence of a food isolate (F6854) from the 1988 episode has previously been determined using automated Sanger sequencing [28]. We sequenced the genome of the corresponding human isolate from the 1988 episode as well as the genomes of a human and a food isolate from the 2000 outbreak. Sequence comparisons of these genomes enabled us to characterize *L. monocytogenes *evolution during short-term survival in a non-controlled non-host environment and to determine the value of full genome sequencing as a subtyping approach in follow-up studies to outbreak investigations.

## Results

### Genome sequencing of three *L. monocytogenes *isolates by 454 pyrosequencing

The human isolate from the 1988 listeriosis case (F6900) and a human (J0161) and a food (J2818) isolate from the 2000 multi-state listeriosis outbreak in the US (Table 1) were sequenced using the 454 GS20 Genome Sequencer (454 Life Sciences, Branford, CT), which uses the "sequencing by synthesis" technology [29]. Chromosomal sequences ranged from 2.96 Mb to 2. 97 Mb, the sequencing depth ranged from 24 to 29.15-fold. The sequenced genomes captured between 93.79 and 96.08% of the EGD-e reference genome (Table 2).

**Table 1:** Human and food isolates from the 1988 sporadic listeriosis case and the 2000 outbreak used for genome comparisons

Isolates	Date sample/specimens was collected	Source	Genome sequencing method used	Source of genome sequence
F6854^(1)^	December 1988	Food	Sanger	[28]
F6900	December 1988	Human	454	This study
J2818	Fall 2000	Food	454	This study
J0161	October 2000	Human	454	This study

^(1)^The F6854 genome sequence represents one pseudomolecule in which gaps between the contigs were closed with random sequences using the fully sequenced genome of EGD-e as a reference [28].

**Table 2:** Assembly quality metrics for strains sequenced in this study

Strain	F6900	J0161	J2818	FSL J1-194
Contig N50^(1)^	434 kb	148 kb	167 kb	156 kb
Total contigs	35	49	38	43
Assembly size	2.96 Mb	2.97 Mb	2.97 Mb	2.93 Mb
Aligned sequence length (nt)^(2)^	2,921,655	2,921,243	2,921,377	-
*comK *prophage sequence length (number of contigs)^(3)^	38,876 bp (5)	41,349 bp (4)	42,001 bp (2)	42,189 bp (1)
Contigs to capture 90% of assembly	11	19	19	19
Fraction bases Q40^(4)^	99.5%	99.7%	99.8%	99.7%
Fraction reference covered	95.97%	95.97%	96.08%	93.79%
Assembled coverage	25×	30×	24×	22×

^(1) ^Length-weighted median that describes the contig size in which at least half the bases reside.

^(2) ^Length of each sequence in the refined alignment containing the 1988 and 2000 isolates. The length for F6854 is 2,921,388 nt.

^(3) ^Sequenced length of the prophage inserted into *comK *for each isolate/strain. The length for F6854 is 41,259 nt.

^(4) ^As reported by 454 assembly software.

In addition to the chromosomal sequences, a plasmid sequence was found in the 2000 human isolate (J0161). This plasmid represented two non-overlapping contigs of 69,915 bp and 12,763 bp. These two plasmid contigs showed a very high level of nucleotide identity (>99%) with pLM80, an approx. 80 kb plasmid previously reported in the serotype 4b (lineage I) strain H7858 [28]. The two plasmid contigs included all 95 ORFs previously identified in pLM80 [28]. Genes found in the plasmid contigs included at least 11 genes annotated as transposon genes in pLM80 and two genes associated with cadmium resistance. The observation that this plasmid was only sequenced in the 2000 human isolate (J0161) suggests either a loss of the plasmid in the other isolates (during propagation in the food processing plant, the food or the human host for the 1988 isolate or during isolation) or an acquisition of this plasmid in the 2000 human isolate.

### Genome alignment and polymorphism analyses

**Figure 1:**
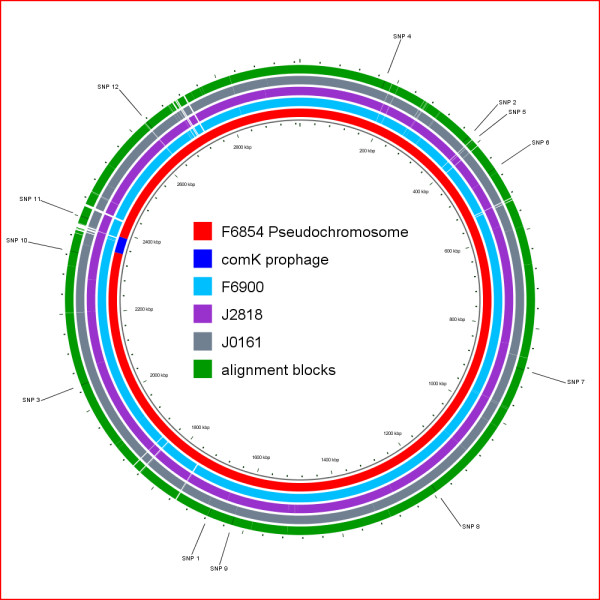
**Circular map of genomes analyzed**. The F6854 pseudochromosome is shown in red except for the *comK *prophage which is shown in blue. The genomic contigs of F6900, J0161 and J2818 are shown in blue, purple and gray, respectively. The blocks of aligned sequence in the refined alignment of F6854, F6900, J0161 and J2818 are shown in green. All sequences were aligned against the pseudochromosome of F6854, which was used as the reference.

To identify genomic differences between the human and food isolate from the 1988 case as well as a food and human isolate from the 2000 outbreak, we aligned the 4 genomes using TBA (Threaded-Blockset Aligner) [30] and refined this alignment using MUSCLE [31]. The refined alignment of the four genomes had a total length of 2,922,773 bp (including gaps) (Figure 1). The aligned sequences for each genome ranged from 2,921,243 to 2,921,655 (Table 2). As between 1.2% and 1.7% of the genome sequences for each isolate could not be included in the alignment, it is possible that the four isolates contain some differences in addition to those detailed below. Regions not included in the alignment most likely represent regions not sequenced in one or more genomes, deletions/insertions, or genome fragments replaced by a non-homologous sequence (i.e., within the *comK *prophage). Specifically, while the length of the *comK *prophage sequence ranged from 38,876 to 42,001 bases in the four isolates (Table 2), the *comK *prophage alignment (including gaps) was only 28,886 bp, indicating extensive diversification including presence of non-homologous prophage sequences in the different isolates (as described in detail below). This difference represents 25% of the non-aligned sequences in the whole genome alignment, although this prophage represents less than 2% of the genome size.

The final alignment was divided in three distinct non-overlapping alignments representing (i) the *comK *prophage (28,886 bp), (ii) the tRNA Thr-4 prophage (34,703 bp), and (iii) the backbone sequence (2,859,184 bp) representing the rest of the chromosomal sequence. Identification of polymorphisms and recombination were carried out separately for each alignment. The *comK *prophage alignment had 1,274 polymorphic sites that differentiated the two 1988 isolates from the two 2000 isolates; at all of these sites, the isolates from the same year were identical to each other. In contrast, the tRNA Thr-4 prophage alignment had one confirmed polymorphic site, which differentiated the 1988 isolates from the 2000 isolates; this site represents a nonsynonymous transversion (Table 3). Surprisingly, besides the polymorphisms found in the *comK *prophage, the polymorphism in the tRNA Thr-4 prophage represents the only SNP that differentiates the 1988 isolates from the 2000 isolates.

**Table 3:** Confirmed single nucleotide polymorphisms found among clinical and food isolates associated with the 1988 sporadic listeriosis case and the 2000 listeriosis outbreak

**SNP no.**	**Position**^**(1)**^	**Locus** **(LMOf6854)**^**(2)**^	**Annotation**	**Nucleotide change**	**Amino acid change**
**SNPs unique to isolate F6900 (1988 human isolate)**					
1	1675779	1688 ↔ 1688.1	*trpE*, anthranilate synthase component I ↔ alcohol dehydrogenase, iron-containing	A → G	Noncoding
**SNPs unique to isolate J0161 (2000 human isolate)**					
2	378489	0351 → 0352	nikkomycin biosynthesis domain protein → N-acetylmannosamine- 6-phosphate epimerase	C → T	Noncoding
3^(3)^	2053116	2071 ← 2072	ABC transporter ← DNA-binding response regulator	T → C	Noncoding
**SNPs unique to isolate J2818 (2000 food isolate)**					
4	174721	0150 → 0150.1	oligopeptide ABC transporter → conserved hypothetical protein	T → C	Noncoding
5	392427	0365.1 → 0367	conserved hypothetical protein → *inlI*, cell wall surface anchor family protein	G → A	Noncoding
6	462089	0447	Phosphoenolpyruvate synthase	G → A	113Pro→113Ser ^(4)^
7	885898	0876 ↔ 0877	Membrane protein ↔ transposase OrfA, IS3	A → G	Noncoding
8	1193390	1203	*pduP*, CoA-dependent propionaldehyde dehydrogenase	C → A	359Pro→359Thr^(5)^
9	1625104	1635.1	RDD family	G → T	2Ala → 2Asp^(5)^
10	2327842	2332	*addB*, ATP-dependent nuclease, subunit B	C → A	388Trp→388Cys^(5)^
11	2401731	2435.1	Unknown function	G → A	Synonymous
**SNP differentiating the two 1988 isolates from the two 2000 isolates**					
12	2635720	2659	Phage tail component, N-terminal domain	A → T	419Ile→419Leu^(5)^

^(1)^Nucleotide position in the full alignment of F6854, F6900, J0161 and J2818; this alignment is available at

^(2)^Arrows indicate direction of genes in the chromosome, e.g. 0351 → 0352 means that the polymorphism falls downstream to LMOf6854_0351 and upstream to LMOf6854_0352. ↔ means that the polymorphism falls upstream to both genes (possible of genes are transcribed divergently).

^(3)^SNP #3 is not present in FSL R2-499, the J0161 subculture sent from CDC to the Cornell Food Safety Laboratory (FSL), and is also not present in nine other human isolates linked to the 2000 listeriosis outbreak, which were also tested; this SNP thus appears to have arisen during laboratory passage of the J0161 clone sequenced at the Broad Institute.

^(4)^This nonsynonymous change falls within the phosphoenolpyruvate/pyruvate binding domain (aa 3 to aa 341)

^(5)^These nonsynonymous changes fall into regions that have no specific predicted domains

Among the 11 confirmed polymorphisms in the backbone sequence (excluding the *comK *and Thr-4 prophage) of the J0161 subculture sequenced at Broad, eight were specific to J2818 (2000 food isolate), one was specific to F6900 (1988 human isolate), and one was specific to J0161 (2000 human isolate) (Table 3). In addition, one polymorphism (SNP #3; Table 3) was present in the J0161 subculture sequenced at Broad, but was not found in the J0161 subculture used for re-sequencing at Cornell (see "Methods"). Among the eight polymorphisms specific to the 2000 food isolate (J2818, Table 3), three fall in intergenic regions and five fall in coding sequences, including one representing a synonymous change and four representing nonsynonymous changes (Table 3). The 2000 human and food isolates thus differ from each other in four nonsynonymous sites, including one substitution (SNP 10; Table 3) in *addB*, which encodes a protein that is part of the enzyme AddAB [32,33]. AddAB is analogous to *E. coli *RecBCD, which is responsible for processing double-stranded breaks in the chromosome [33,34].

### Distribution of the human outbreak isolate specific SNPs and plasmid sequences among other outbreak associated human clinical isolates

As the 2000 human and food isolates differed by 9 SNPs, we identified additional human isolates associated with the 2000 outbreak to validate these SNPs; unfortunately no additional food isolates associated with this outbreak were available for SNP validation. Nine isolates with ribotype DUP-1053A (representing the ribotype of the 1988 and 2000 food and human isolates) obtained between August and November of 2000 from human clinical cases in Ohio, Michigan and New York were found to be slow rhamnose-fermenters. These isolates were also found, by PFGE using *Apa*I and *Asc*I for restriction of DNA, to be indistinguishable from each other and from the 1988 and 2000 human and food isolates sequenced. As these results strongly suggested that these nine isolates were from humans that were infected as part of the 2000 outbreak, we screened these isolates for the 12 confirmed polymorphisms identified among the genome sequences for the human and food isolates from the 1988 case and the 2000 outbreak as well as for the presence of two plasmid-specific sequences. All 9 isolates were positive by PCR for both plasmid sequences, indicating that the plasmid found in J0161 is present in most other human isolates associated with this outbreak. None of these nine human isolates had any of the polymorphisms specific to F6900 (1988 human isolate) or J2818 (2000 food isolate).

Furthermore, none of the nine isolates shared the one SNP specific to the subculture of the 2000 human isolate (J0161) that was sequenced at Broad (SNP 3; Table 3), but that was not found in the J0161 subculture that was used for re-sequencing at Cornell, supporting the hypothesis that this polymorphism arose during laboratory passage of the J0161 subculture sequenced at Broad. Nevertheless, all nine human isolates showed (i) nt sequences identical to J0161 (the sequenced 2000 human isolate) at the eight sites that carry unique SNPs in the 2000 food isolate (J2818), (ii) the SNP unique to the 2000 human isolate J0161 (SNP 2; Table 3) as well as (iii) the tRNA Thr-4 prophage SNP (SNP 12; Table 3) that differentiates the 1988 isolates from the 2000 isolates. SNP 2 thus does not represent a random mutation unique to the sequenced human isolate from the 2000 outbreak (e.g., mutation that arose during passage or infection), but was found in all human clinical isolates surveyed from this outbreak.

### Intracellular growth assays of the 2000 human and food isolates

While it is unlikely that any of the few nonsynonymous substitutions observed would affect the fitness of *L. monocytogenes *strains tested here, we performed preliminary experiments to test whether the nonsynonymous sites differentiating the 2000 human and food isolates (may affect the ability of the 2000 human and food isolates to multiply inside macrophage cells. We specifically surmised that the nonconservative Trp → Cys change in *addB *(i.e., SNP 10, Table 3) could alter the function of the enzyme AddAB [32,33], which is analogous to *E. coli *RecBCD [33,34]. As RecBCD deficient *E. coli *mutants have been shown to be more susceptible to oxidative DNA damage [35]; we hypothesized that the 2000 food and human isolates may differ in their ability to survive inside activated macrophages (i.e., J774 cells stimulated with LPS) where they may be exposed to oxidative stress. We used FSL R2-499 (a subculture of J0161 confirmed as having SNP 10 and thus encoding the same *addB *allelic variant as J0161) and the 2000 food isolate J2818 for these experiments. As no apparent difference in intracellular growth of these two strains was observed when the strains were initially characterized in separate intracellular growth assays (Additional file 1), we performed triplicate competition experiments by infecting J774 cells with both strains simultaneously; competition assays like this typically provide for more sensitive means to identify whether one strain has a competitive advantage over another strain in a given environment [36,37]. In the competitive intracellular growth assay the relative recovery of both isolates after 9 h of intracellular growth did not differ significantly (*P *> 0.05; chi-square test); overall, 16 and 14 colonies, respectively, with the J2818 and the J0161 *addB *allelic type were recovered (3/10, 7/10, and 6/10 isolates carried the J2818 *addB *allelic type in replicates 1, 2, and 3, respectively). While these data suggest that the mutations that differentiate the two 2000 isolates do not affect the intracellular growth capabilities, of these isolates, in activated J774 macrophage cells, future phenotypic and animal experiments would be needed to further test whether the 2000 human and food isolate genotypes differ in their fitness. As no data on the effect of an *L. monocytogenes addB *null mutation are available, it is unclear which physiological functions may be affected by nonsynonymous mutations in this gene. Future experiments thus should involve characterization of an *addB *null mutant before conducting additional phenotypic and animal experiments to determine the effect of the observed nonsynonymous mutation in *addB *and/or other nonsynonymous mutations differentiating the 2000 human and food isolates.

### Estimation of congruence between observed low mutation rate and known mutation rates in bacterial populations

Overall, we only found a single synonymous nucleotide difference (SNP 11; Table 3) between the 2000 food isolate (J2818) and the 1988 isolates and no synonymous nucleotide difference between the 2000 human isolate (J0161) and the 1988 isolates. In order to determine whether this low number of synonymous changes is consistent with previously reported mutation rates for bacteria, we calculated the estimated number of generations between the 1988 isolates and the 2000 food isolates (J2818) using previously reported mutation rates [2,3,38], including 3.30 – 4.50 × 10^-10 ^per bp per generation for experimental *Myxococcus xanthus *populations [3] and 1.44 × 10^-10 ^per bp per generation for experimental *E. coli *populations [2]. Ochman *et al*. [38] estimated a mutation rate of 4.50 × 10^-9 ^per bp per year (approximately 2.25 × 10^-11 ^per bp per generation assuming ~200 generations per year) for natural populations of *E. coli *and 8.20 × 10^-9 ^per bp per year (approximately 2.05 × 10^-10 ^per bp per generation assuming 40 generations per year) for natural populations of *Buchnera*. The number of generations was calculated as the (number of synonymous substitutions, i.e., 1])/[(number of synonymous sites, i.e., 5.65 × 10^5^) × (mutation rate)]. Using the two extremes of the suggested mutation rates (2.25 × 10^-11 ^and 4.50 × 10^-10 ^per bp per generation) we estimated that between 3,933 and 78,666 generations separated the 1988 isolates from the 2000 isolates. Assuming a 12 year time span between the two sets of isolates, these mutation rates translate into 328 and 6,556 generations per year (0.90 – 17.96 generations per day), yielding a generation time ranging from 1.34 to 26.67 hours. The generation time estimate (1.34 h) based on the low mutation rate (2.25 × 10^-11 ^mutations per bp per generation) proposed for natural populations of *E. coli *[38] is only about 2-fold longer than the estimated generation time for *L. monocytogenes *in rich media broth at 30°C (0.7 h) [39].

Other *L. monocytogenes *generation times reported were 14.2 h (in rich-media at 4°C [39]) and 111.3 h (for *L. monocytogenes *in soil exposed to temperatures fluctuating between -29°C to 12°C [40]). We thus propose that a mutation rate closer to 4.50 × 10^-10 ^(as estimated from an experimental population of *M. xanthus *[3]) is more likely to represent the mutation rate for *L. monocytogenes*, as a generation time of approx 24 – 28 h seems plausible for *L. monocytogenes *in natural environments. Under this mutation rate, generation time, and assuming a Poisson distribution, the probability of observing zero or one synonymous substitution is roughly the same (~36.8%), which is consistent with the observation that the 2000 food isolate (J2818) differed from the 1988 isolates by one synonymous change, while the 2000 human isolate (J0161) showed no synonymous changes as compared to the 1988 isolates. In conclusion, the low rate of mutations observed for the isolates studied here is thus clearly within the range expected based on previously reported mutation rates for other bacteria.

### Comparative analyses of comK prophage sequences in the 1988 and 2000 isolates

As the *comK *prophage in the 2000 isolates showed considerable divergence from the *comK *prophage in the 1988 isolates (1,274 nt differences between the 1988 and 2000 prophage sequences), further analyses were performed on the *comK *prophage sequences. Initial BLAST searches against (i) the F6854 genome, (ii) genome sequences deposited in GenBank [41], and (iii) genome sequences in the *L. monocytogenes *database at the Broad Institute [42] found that the following prophages showed the highest similarity to the 2000 isolate prophage sequence: (i) the *comK *prophage sequence in *L. monocytogenes *FSL J1-194 (BLAST score of 2.23 × 10^4 ^bits), (ii) the *comK *prophage in F6854 (the 1988 human isolate; 1.88 × 10^4 ^bits), (ii) the phage A500 (1.03 × 10^4 ^bits) (GenBank accession DQ003637), (iii) the *comK *prophage in EGD-e (1.01 × 10^4 ^bits) [43], and (iv) the phage A118 (8.82 × 10^3 ^bits) [44]. Additionally, BLAST searches of the individual genes in the *comK *prophage against these same databases, showed that 35 of the 65 genes in the 2000 isolates showed the best match with genes in the FSL J1-194 prophage (Additional file 2); 14 and 11 genes in the 2000 isolates showed the best match with genes from the phage A118 and the EGD-e *comK *prophage, respectively. While these data suggest that diversification of the *comK *prophage in the 2000 isolates does not represent a prophage replacement with a sequenced prophage, replacement could have occurred with a prophage not represented in the available nucleotide sequence databases.

**Figure 2:**
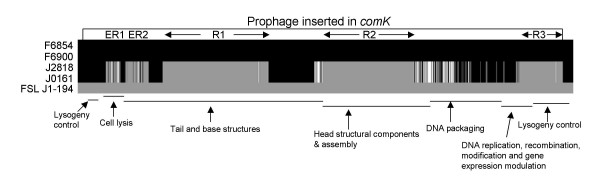
**Schematic of recombination events in *comK *prophage**. The figure represents an alignment of the 1892 polymorphic sites in the *comK *prophage (including coding and non-coding regions) as well as 13 polymorphic sites immediately upstream the prophage and 41 polymorphic sites immediately downstream of the prophage. Sites representing nt residues found in F6854 and F6900 (1988 isolates) are shown in black and sites representing nt residues found in FSL J1-194 (a *L. monocytogenes *serotype 1/2b isolate with the *comK *prophage most closely related to the prophage found in the 2000 isolates) are shown in gray. The nt sites in J0161 and J2818 (2000 isolates) are shaded so that nt identical to the 1988 isolates are in black, nt identical to FSL J1-194 are in gray, and nt that match neither the 1988 isolates nor FSL J1-194 are in white. Apparent darker shades of gray in this figure represent adjacent sites with black and gray or white. R1, R2, and R3 (recombination 1 to 3) represent three main recombinant blocks that were likely introduced from a lineage I strain similar to FSL J1-194 into a descended of the 1988 isolates, yielding the 2000 isolate genotype. The two shorter mixed gray and white blocks at the 5' end, marked as ER1 and ER 2 (ER = external recombination) are likely to have been introduced from a *L. monocytogenes *genotype not included in this alignment ("external") into a descendent of the 1988 isolates.

To determine whether recombination events could have contributed to the diversification of the *comK *prophage, recombination analyses were performed on an alignment of the four *comK *prophage sequences for the 1988 and the 2000 isolates and the prophage most closely related to the *comK *prophage in the 2000 isolates (i.e., the *comK *prophage in isolate FSL J1-194). Sawyer's test provided evidence for recombination among the five prophage sequences in the alignment (i.e., inner fragments, *P *< 0.0001) as well as for recombination with sequences not included in this alignment (i.e., outer fragments, *P *< 0.0001). Overall, five inner recombination events involving the *comK *prophage in the two 2000 isolates and FSL J1-194 were initially identified. Visual analysis of the prophage sequences suggests at least three independent recombination events in the two 2000 isolates, relative to the two 1988 isolates; for these three recombinant regions (marked as R1, R2, and R3 in Figure 2) the prophage sequences in two 2000 isolates were more similar to FSL J1-194 than to the prophage sequences in the two 1988 isolates (Figure 2). Analyses of nt identities between the 1988 and 2000 isolates and FSL J1-194 for all individual ORFs in the *comK *prophage (Additional file 2), also supported that extensive recombination occurred in this prophage. While 15 genes showed 100% identity between the 1988 and 2000 prophage genomes (e.g. LMOf6854_2341.9; Additional file 2), 24 other genes showed identities < 95% between the 1988 and 2000 prophage genomes and 100% identity between the 2000 isolates and FSL J1-194 (e.g. LMOf6854_2352.2; Additional file 2), suggesting introduction of these genes into the ancestor of the 2000 isolates from a phage or prophage sequence closely related to that found in FSL J1-194. Eleven prophage genes in the two 2000 isolates showed < 98% identity to the corresponding prophage sequence in the 1988 isolates and a best match with a sequence other than that of FSL J1-194 (e.g. LMOf6854_2371; Additional file 2), suggesting introduction from a distinct phage or prophage genotype.

For 18 genes present in the *comK *prophage of the 1988 isolates (i.e., F6854 and F6900), no homologues were identified in the genomes of the 2000 isolates; 15 of these genes appear to have been replaced in the 2000 isolates by non-homologous genes (Additional file 2). These deletions and replacements in the 2000 isolates are likely consequences of recombination events that integrated or replaced large sequences, as supported by the observation that some observed gene deletions or replacements were located in large recombinant fragments identified by Sawyer's test. For example, the eight adjacent genes LMOf6854_2352.2 to LMOf6854_2360.1 found in the 1988 isolates appear to have been replaced by a block of seven genes in the 2000 isolates (Sawyer's tests identified a recombinant fragment, which corresponds to this replacement event). Two of these eight genes present in the 1988 isolates (LMOf6854_2352.6 and LMOf6854_2352.7) show no homology to the genes present in the 2000 prophage (Additional file 2).

## Discussion

Determination of genome sequences for three *L. monocytogenes *isolates obtained from foods and human listeriosis cases linked to a single food processing facility, in conjunction with a previously reported genome sequence for a food isolate linked to the same facility [28], provided a unique resource to gain insight into the short-term evolution of *L. monocytogenes *in non-controlled environments. Overall, our data indicate that short term evolution of *L. monocytogenes *in natural non-controlled environments involves limited diversification of the genomic backbone, but can involve considerable phage-mediated diversification as well as plasmid loss or gain. In addition, our genome sequencing data support that a human listeriosis outbreak that occurred in 2000 in the US was caused by a *L. monocytogenes *strain that is likely to have persisted in a food processing plant over at least 12 years, supporting the utility of genome sequencing for high resolution follow-up studies in disease outbreak investigations.

### Short term evolution of *L. monocytogenes *in natural environments can involve considerable phage mediated diversification and loss or gain of plasmids

While a few singletons (i.e., single base differences found in only one isolate) were observed in the genome backbone of the two 1988 and the two 2000 isolates, the only polymorphisms that differentiate the 1988 isolates from the 2000 isolates fall within the two prophages inserted into *comK *and tRNA-Thr-4. While the tRNA-Thr-4 prophage region showed only one SNP that differentiated the 1988 and 2000 isolates, the *comK *prophage region differs considerably between the two 1988 and the two 2000 isolates. Our data suggest that multiple recombination events lead to diversification of the *comK *prophage, although the occurrence of a single prophage replacement event cannot be excluded. Recombination events seem to have replaced a number of phage genes (including several genes that were annotated as encoding proteins with essential roles in the lytic cycle) with allelic variants or non-homologous genes. For example the gene encoding the major capsid in the 1988 isolates (LMOf6854_2352.7) was replaced with a non-homologous major capsid gene (LMPG_02241.2) in the 2000 isolates. Mechanistically, the observed recombination events in the *comK *prophage most likely occurred through infection with one or more phages in one or more cells of the 1988 genotype, followed by subsequent recombination or phage replacement. In addition to diversification of the integrated prophage genomes observed here, it has been well documented that phages can be vectors for horizontal transfer of genes located in the genome backbone [45] and that phages are capable of generalized transduction in *L. monocytogenes *[46]. While we did not find any evidence for horizontal gene transfer outside the prophage region in the isolates studied here, horizontal gene transfer in genes in the backbone genome has been well documented in *L. monocytogenes *[47-49]. While very little is known about the frequency of phage insertion and phage transduction in bacteria in natural environments, Hodgson [46] reported that rates of phage transduction of *Listeria *under laboratory conditions ranged from about 1 to 300 transductants per 10^7 ^PFU. In conclusion, while short term evolution of *L. monocytogenes *in natural environments involves limited diversification of the genomic backbone, phage-mediated genetic changes appear to be major mechanisms for diversification and evolution of *L. monocytogenes *during short evolutionary time frames. This is particularly interesting as there is no evidence for phage-encoded virulence genes in *Listeria*, unlike for a number of other pathogens where phage mediated transfer and diversification of virulence genes appear to represent an important mechanism for diversification.

Our data are consistent with the overall idea that bacteriophages play multiple important roles in the evolution of bacterial populations in natural environments, including (i) providing selective pressure on bacterial populations (e.g., [50-52]) and (ii) providing a mechanism for rapid horizontal exchange of genetic material (e.g., [53]). Contributions of bacteriophages to genetic diversification also have been well characterized in different pathogenic and non-pathogenic bacterial species. For example, the *V. cholerae *CTX genetic element represents the genome of a lysogenic bacteriophage, which carries the genes for cholera toxin [53]. This lysogenic phage can be induced in toxigenic strains and the resulting phage particle appears to be able to transduce non-toxigenic *V. cholerae *[54]. Similarly, in different *E. coli *pathotypes, shiga-toxin genes as well as genes encoding other virulence factors are located on different prophages [55]. Diversification of prophages integrated in the chromosome of bacteria also appears to occur rapidly and commonly and contributes to bacterial genome evolution. For example, in enterohemorrhagic *E. coli *considerable diversification of prophages carrying virulence determinants has been observed, likely indicating independent infections of host bacteria by different bacteriophages carrying these virulence determinants [55]. Similarly, considerable diversity has been observed in the genome of the *Vibrio *CTX prophage (e.g., [56]). The importance of bacteriophages and lysogenic prophages in the evolution of gram-positive pathogens has also been documented (e.g., [57]). For example, the rapid emergence of serotype M3 group A *Streptococcus *(GAS) has been associated with the acquisition of a prophage that contained a unique combination of virulence genes, which was probably generated through several recombination events [58]. Moreover, most of the genetic diversity among GAS strains seems to be phage-related [59,60]. Prophages and diversification of prophage-associated genes thus appears to play an important role in the evolution of various bacterial species.

In addition to the diversification in the *comK *prophage, we also found that the four isolates differed in their plasmid content; a plasmid sequence was only found in the 2000 human isolate, as well as in nine additional human isolates linked to this outbreak. While our findings suggest that plasmid loss or acquisition events occurred, it can not be determined whether these changes occurred during *L. monocytogenes *survival in the processing plant, during passage of isolates, or during human infection. Rapid diversification of *L. monocytogenes *due to plasmid loss or acquisition has previously been reported and isolates from the same outbreak have been shown to differ in their plasmid profiles [21]. However, it is important to note that the media commonly used to isolate *L. monocytogenes *(particularly media used for isolation from food samples) contain acriflavine, a plasmid curing agent [61,62]. Therefore, it is possible that the isolates for which no plasmid sequence was obtained lost this plasmid during isolation or during laboratory passage.

### Genome sequence data support that a human listeriosis outbreak in 2000 was caused by a *L. monocytogenes *strain that persisted in a food processing plant

Our data showed that the human and food isolates from a sporadic case in 1988 and the human and food isolates from an outbreak in 2000, which were linked to the same food processing facility, had genome sequences that showed no major inversions, deletions and insertions, although minor changes like these cannot be ruled out since the genome sequences have not been fully finished and assembled. While the four isolates showed a total of 12 confirmed SNPs, all 11 SNPs that occurred in the genome backbone represented singletons, i.e., they were found in only one of the four isolates. The only mutations that differentiated the 1988 isolates from the 2000 isolates were (i) one single SNP in the tRNA-Thr-4-prophage and (ii) a large number of SNPs and deletions in the *comK *prophage, which most likely represent multiple recombination events. This high level of overall genome conservation between the human and food isolates from the 1988 listeriosis case and the 2000 outbreak strongly supports that these two incidences were caused by the same *L. monocytogenes *strain, which is likely to have persisted over at least 12 years in the processing plant that produced the food linked to both the 1988 case and the 2000 outbreak. Persistence is also supported by the observation that the PFGE type represented by the persistent strain seems rare (it was only found once among 495 food isolates characterized by PFGE [22]). Re-introduction from the immediate environment surrounding the plant is a possible, but unlikely, alternative to persistence of the specific strain in the processing plant, particularly as ready-to-eat meat processing plants will have controls in place to minimize introduction of microorganisms. Persistence in turkey flocks providing raw materials for this plant is even less likely as the heat treatment used for production of frankfurters and deli meats inactivates *L. monocytogenes *[63]. Similarly, re-introduction through a persistently infected worker is highly unlikely as people rarely shed *L. monocytogenes *[64,65]. Importantly, even in the unlikely scenario that the unique strain linked to listeriosis cases in 1998 and 2000 was reintroduced, rather than having persisted in the plant, the isolates characterized here would still represent a set of closely related isolates with a parent-descendant relationship, which have multiplied in a non-controlled environment over about 12 years. Thus, the alternative scenarios outlined above do not affect the main conclusions of our study, including that phage mediated mechanisms are critical for short term diversification of *L. monocytogenes*.

Overall, our data demonstrate the utility of using full genome sequencing for follow-up investigations to epidemiological and source tracking studies. In particular, full genome sequencing clearly allowed for differentiation of the *L. monocytogenes *isolates from the 1988 case and the isolates associated with the 2000 outbreak, even though these isolates were previously found to be identical by other subtyping methods, including PFGE [26]. Genome sequencing not only represents the most sensitive subtyping approach conceivable, but also provides for specific identification of differences between isolates, which can be used to assess whether differences between two isolates are likely to have occurred within a short time frame or not. This assessment is critical for source tracking as genomic diversification that can lead to different genomic restriction profiles can clearly occur over short time periods (e.g., through diversification of prophages, loss of plasmids). Rapid diversification in certain hot spots (e.g., prophages) represents a challenge as the current gold standard method for bacterial subtyping is a genomic restriction profiling approach (i.e., PFGE [66]), which means that closely related isolates may have different PFGE types (as seen in some outbreaks (e.g., [21,67]). Importantly though, the standard restriction enzymes used for *L. monocytogenes *PFGE analyses (i.e., ApaI and AscI [68]) only interrogate <500 nt for SNPs; based on the genome sequence for F6854, there are 39 restriction sites for ApaI, which recognizes a 6 bp motif (yielding 234 bp of DNA interrogated by this enzyme), and 15 restriction sites for AscI, which recognizes a 8 bp motif (yielding 120 bp of DNA interrogated by this enzyme). Thus, genetically distinct isolates may share the same PFGE pattern. For example, Nightingale et al. [69] found that *L. monocytogenes *isolates with the same PFGE pattern may differ by presence/absence of premature stop codons in a number of genes, including the virulence gene *inlB*. In contrast to PFGE, our sequence analyses used >2.9 Mb of aligned sequences for subtype discrimination, thus providing for vastly improved detection of substitutions. While we cannot exclude that the isolates characterized show differences in their genomes in addition to those reported here (as the genomes have not been closed and include gaps), this approach clearly provides for a comprehensive sampling of the genomic diversity in the characterized isolates. Future use of full genome sequencing for epidemiological investigations of bacterial infectious disease outbreaks thus seems feasible assuming anticipated continued decreases in the cost of DNA sequencing [70] and availability of computational tools that allow for rapid and reliable genome assembly and comparisons.

### Isolate specific SNP patterns in the genomic backbone may occur during environmental growth, isolation or subculture

**Figure 3:**
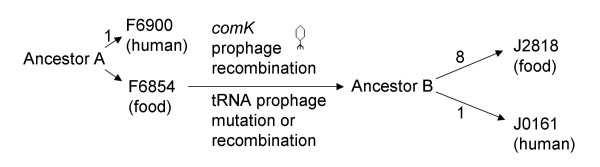
**Schematic of the putative evolutionary history of the *L. monocytogenes *strain in the food facility between 1988 and 2000**. Numbers on the arrows represent new mutations. Ancestor A is the ancestor of F6854 and F6900 (the food and human isolate, respectively, from the sporadic case in 1988) and Ancestor B is the ancestor of J0161 and J2818 (the food and human isolate, respectively, from the outbreak in 2000).

Analysis of the single nucleotide polymorphism in the genome backbone of the isolates from both the 1988 listeriosis case and the 2000 listeriosis outbreak also provided us with the ability to probe the evolution of *L. monocytogenes *isolates during environmental persistence and during human infection. Based on our data, we hypothesize that the ancestor of F6854 and F6900 (1988 isolates) contaminated the plant in or before 1988. The single SNP differentiating the 1988 human isolate from the 1988 food isolate is consistent with a very close ancestral relationship between these two isolates; the unique SNP in the human isolate could have occurred in the ancestor of the human isolate (F6900) during multiplication in either the plant, the contaminated food, or the infected human or during isolation and propagation of the isolate. The ancestor of F6854 remained in the plant and persisted until 2000. Between 1988 and 2000, the *comK *prophage of that strain was replaced (through one or several recombination events) by the prophage found in the 2000 isolates. Moreover, one mutation that differentiates the 1988 isolates from the 2000 isolates occurred in the tRNA prophage (possibly through a substitution or due to a recombination event). After the *comK *prophage replacement and the tRNA mutation, one mutation in J0161 (the 2000 human isolate) and eight mutations in J2818 (the 2000 food isolate) occurred and differentiated these two isolates from each other and from the 1988 isolates. The substitution that is unique to the 2000 human isolate must have occurred during growth prior to infection and isolation as it was found in the sequenced human isolate (J0161) as well as in an additional 9 human isolates (see Figure 3). On the other hand, the substitutions in the 2000 food isolate could have occurred during multiplication in the plant, the contaminated food, or during isolation and laboratory propagation. The observation that the 2000 food isolate carries eight unique SNPs, which clearly separated this isolate from the other three isolates, while carrying a *comK *prophage region identical to the 2000 human isolate, suggests that these mutations occurred after the two 2000 isolates diverged from the 1988 strain. The possibility that these SNPs arose during laboratory passages is supported by our observation that two subcultures of the 2000 human isolates differed by one SNP as well as observations that two subcultures of *Bacillus anthracis *strain Porton differed by a number of SNPs [71].

## Conclusion

Analyses of full genome data of four *L. monocytogenes *isolates associated with a sporadic human listeriosis case (in 1988) and a subsequent outbreak (in 2000), both linked to the same food processing facility, not only demonstrated the utility of full genome sequencing for detailed follow-up studies that can provide an improved understanding of infectious disease transmission, but also represent one of the few datasets that provide insights at the full genome level into evolutionary patterns of bacteria in non-controlled environments. The limited number of mutations observed in the genome backbone are consistent with mutation rates in bacteria previously calculated using data from both experimental (e.g., [2,3]) and natural populations [38]. Rather than single base-pair mutations or rearrangements in the genomic backbone, diversification in prophage sequences, and possibly plasmid loss and acquisition (as also supported by data from others [21]), seem to represent the major drivers of diversification in *L. monocytogenes *in non-controlled environments. Interestingly, in *L. monocytogenes*, diversification by these mechanisms seems to not target virulence genes as neither plasmid nor phage-based *L. monocytogenes *virulence genes have been identified so far. The clearly observed pattern of considerable phage-mediated diversification in highly related *L. monocytogenes *isolates with nearly identical genome backbone sequences also demonstrates that the absence of any possibility for phage-mediated diversification during in vitro evolution studies of bacteria represents a major constraint associated with these studies. This report is one of the first studies on the short-term genome evolution of bacteria in a non-controlled environment, thus providing important information that may help in interpretation of data from laboratory evolution experiments. Despite the fact that studies on naturally evolving populations in non-controlled environments will necessarily include a number of drawbacks (e.g., difficulty in identifying parent-descendant pairs with a specific number of separating generations), these studies are a critical complement to laboratory-based studies and are needed to improve our understanding of the evolution of bacteria in natural environments.

## Methods

### *L. monocytogenes *isolates

The primary isolates characterized here were a human and a food isolate from a 1988 sporadic listeriosis case in the US [24] (isolates F6900 and F6854; Table 1) as well as a human and a food isolate from a 2000 listeriosis outbreak in the US [25] (isolates J0161 and J2818; Table 1). Isolates F6900, J0161 and J2818 used for preparation of DNA for genome sequencing were directly sent from the US Centers for Disease Control and Prevention (CDC) to Anza Therapeutics, Inc. for genomic DNA isolation. Subcultures used for re-sequencing (to confirm selected polymorphisms) and for tissue culture were independently provided to Cornell from the CDC. At the CDC, isolates were stored at -70°C in sheep's blood (isolate F6900) or in trypticase soy broth with 20% glycerol (J0161 and J2818). At Cornell, all isolates were stored at -80°C in brain heart infusion broth with 15% glycerol.

F6854, the food isolate from the 1988 listeriosis case (Table 1) was isolated from direct plating of the food sample on lithium chloride – phenylethanol- moxalactam (LPM) agar. While the specific details of the isolation procedures for J2818, the 2000 food isolate, were not available, standard procedures for isolation of *L. monocytogenes *from meat products in 2000 would involve primary enrichment in University of Vermont (UVM) broth and secondary enrichment in Fraser Broth (FB) or Buffered *Listeria *Enrichment Broth (BLEB), followed by plating on Modified Oxford (MOX)(MLG 8.06 and MLG 8A.03, the protocols are available at [72]); all of these enrichment media as well as MOX include acriflavine, a potential DNA modifying and plasmid curing agent [61,62].

Standard protocols for the isolation of *L. monocytogenes *from human patients involve collection of blood or cerebrospinal fluid (CSF) with a syringe or directly into blood culture bottles. The blood culture enrichment is sub-cultured onto appropriate plating media (e.g. trypticase soy agar supplemented with 5% defibrinated sheep's blood) at regular intervals. While human isolate F6900 (Table 1) was isolated via blood culture by this protocol (Swaminathan and Graves, unpublished), the procedure used for isolation of human isolate J0161 is not available. Unfortunately, no specific data on the number of passages for each isolate are available.

### Genome sequencing

High molecular weight genomic DNA was isolated from overnight cultures grown in buffered yeast media. Bacteria were lysed using lysin from phage 10403 [46] that had been cloned and purified as described [73]. Genomic DNA was isolated using QIAGEN genomic-tip 100/G columns per manufacturer's recommendations.

Genome sequences for isolates J0161 (human isolate, 2000 listeriosis outbreak) [25,26], J2818 (food isolate, 2000 listeriosis outbreak) [25,26], F6900 (human isolate, 1998 listeriosis case) [24], and isolate FSL J1-194 (unrelated human isolate from a sporadic case [42] were determined at the Broad Institute using the 454 technology [29]. 454 data were assembled using the 454 Newbler assembler, version v1.0.52.60 [74]. Assembly quality metrics for each of the four genome assemblies are described in Table 2. In all cases, the assembly covers ≥93% of the finished reference and the N50 contig size is at least 148 kb. N50 contig size is a length-weighted median representing the size contig in which the typical base is found.

Whole genome shotgun end sequencing of plasmids with 4 to 10 kb inserts and fosmids with 40 kb inserts was performed by ABI BigDye chemistry and detected on ABI 3730 machines as described previously [75].

### Genome sequences used for analyses

The draft genomes of J0161 (version 1), J2818 (version 2), F6900 (version 2) (NCBI accession nos. AARW00000000.1, AARX00000000.1, and AARU00000000.1), sequenced as described above, as well as the unfinished sequence of F6854 (AADQ00000000.1), obtained from the Comprehensive Microbial Resource [76] were used for all analyses. In addition to the chromosome sequences for these four isolates; we also analyzed the plasmid sequence found in the 2000 human isolate (J0161). The genome sequence of F6854 represented a pseudomolecule in which gaps between the contigs were closed with random sequences using the fully sequenced genome of EGD-e as a reference; all details on the construction of this pseudomolecule were described by [28]. The synthetic sequences introduced to close gaps were deleted from F6854 after the initial genome alignment using TBA (as detailed below) and prior to further manipulations and analyses.

The genome sequence for the lineage I strain FSL J1-194 (version 2) (NCBI accession no. AARJ00000000.1), also determined at the Broad Institute, was used for recombination analyses; like the other genomes used here, the genome of FSL J1-194 has not been fully sequenced and closed.

### Genome alignments

The package TBA (Threaded-Blockset Aligner) [30] was used to align the five genomes (i.e., the 1998 and 2000 human and food isolates as well as FSL J1-194) with the package's default settings. TBA runs a series of blastz alignments (i.e., pairwise sequence alignment) between the genome sequences for each isolate. These alignments are subsequently used to build multiple alignment blocks; each block contains the sequences for the isolates included in a pairwise alignment that had been created in the first step. If, for example, J0161 did not have a specific fragment "A" or if the fragment had not been sequenced in this isolate, J0161 did not appear in the block for fragment "A". Because the genome sequence of four of the five isolates is represented in multiple contigs (Table 1), several blocks were created; each block could not contain more than one contig from a given isolate. The blocks were filtered to keep only those blocks that contained sequences from all isolates analyzed. The F6854 sequence was used as a reference to order blocks such that the first block started at the start of the F6854 sequence and the last block ended at the end of the F6854 sequence. After completion of the alignment, 62 ambiguous sites in the F6854 genome sequence (e.g., R, S, W, etc.) were manually changed to the consensus sequence of the other isolates at the same position (at all of these sites isolates J0161, J2818, and F6900 had the same nt; thus, these manipulations would not underestimate the number of true differences between the 1988 and 2000 isolates or between the food and human isolates). The alignment blocks were subsequently edited using custom perl scripts (developed by the authors) and BioEdit version 7.0.5.2 [77] to create one multiple alignment of the sequences in which the blocks were ordered according to the F6854 chromosomal sequence. This alignment was further refined using the program MUSCLE version 3.6 [31] using a gap open penalty of -75.0, a gap extension penalty of -1.0, and the default settings for all other parameters.

### Genome alignment analyses

The final alignment was separated into three sub-alignments, including alignments for (i) the prophage inserted into *comK*; (ii) the prophage inserted into tRNA-Thr-4; and (iii) the genome backbone sequence without the two prophage sequences. These alignments were initially analyzed using DnaSP version 4.0 [78] to identify polymorphic sites in all three alignments. A perl script was developed to identify positions with gaps in the alignment and to identify which sequences had the gaps.

The number of synonymous sites in the genome of F6854 (i.e., 5.65 × 10^5 ^synonymous sites) was calculated as previously described [3] using all annotated protein coding sequences with the exception of the ORFs present within the two prophages.

### Analysis of comK prophage sequences

The coding sequences for all genes annotated in the *comK *prophage in the 2000 isolates (J2818 and J0161) were used as queries in blastn searches against (i) the genomic sequence of F6854 (1988 food isolate); (ii) the *Listeria *database at the Broad Institute website [42]; and (iii) GenBank [41]. For those *comK *prophage genes present in the 2000 isolates that did not have homologous sequences in the 1988 human and food isolates (F6854 and F6900) in the initial blastn search, the absence of a homologue was confirmed by inspection of the corresponding region of the prophage in F6854 (1988 food isolate).

### Recombination analyses

Recombination analyses were performed using Sawyer's test implemented in GENECONV [79]; GENECONV default settings were used for the analyses with the exception that sites with gaps were not considered and mismatches were allowed within the recombinant fragment (gscale = 3).

### Confirmation of polymorphic sites

All 43 polymorphic sites in the backbone alignment and the single polymorphic site in the tRNA Thr-4 prophage were initially checked by comparison with pre-existing Sanger trace files available from NCBI for isolates J0161, J2818 and F6854. If Sanger trace files were not available, polymorphisms were confirmed by re-sequencing; for this purpose, PCR primers were designed, using the F6854 genome as reference, to amplify the regions flanking the polymorphisms (Additional file 3). A total of 33 pairs of primers were designed since in a few cases multiple polymorphic sites could be amplified in a single fragment. PCR products were purified using Exonuclease I (0.5 U/μl) and Shrimp alkaline phosphatase (0.05 U/μl) (USB, NEB) and sequenced, using standard Sanger fluorescent sequencing methods, at the Core Laboratories Center at Cornell University, USA. Among the PCRs performed for sequencing, five amplified more than one fragment (three amplified two fragments and two amplified three fragments), suggesting that those regions represented repetitive regions in the chromosome. Blastn searches of the target sequences that were used to design these five primer sets against the genomes of F6854 and EGD-e confirmed that the fragments amplified by these primers represented almost identical repetitive sequences and DNA sequencing showed that the polymorphic sites initially identified in these regions were due to misalignments of repetitive sequences. Overall, 11 polymorphisms were confirmed by re-sequencing; importantly, the one SNP in the genome backbone initially identified as differentiating the two 1988 and the two 2000 isolates was not confirmed. Interestingly, one polymorphism in the 2000 food isolate (J0161; SNP #3; Table 3) was not confirmed by re-sequencing, but was confirmed in the trace files available in NCBI, indicating that this polymorphism most likely occurred during laboratory passage of the J0161 subculture sequenced at the Broad Institute (as the sequence at this site in the J0161 subculture at Cornell matched the other 2000 isolate and the two 1988 isolates); we considered this polymorphism as a confirmed polymorphism as it was present in the actual isolate sequenced. In summary, thus, among all 44 polymorphisms identified initially, 12 were confirmed. Among the 32 polymorphism not confirmed, eight represented sequencing errors in F6854 (which was sequenced with the Sanger method) and one represented a sequencing error in J2818; 12 of the polymorphisms that were not confirmed represent problems in the number of bases within homopolymeric tracts and misplacement of the gap in the alignment, and 11 represented misalignments of repetitive regions (Additional file 4).

### Confirmation of selected indels

We also chose genome alignment gaps (representing possible indels) that (i) differentiated the two 1988 isolates from the two 2000 isolates (26 gaps) or (ii) differentiated the two human isolates from the two food isolates (34 gaps) for verification. Gaps were initially verified using Sanger trace files available for isolates J0161, J2818 and F6854 and sequencing by synthesis technologies data files. For a total of six gaps that could not be verified either due to ambiguous sequence data or lack of Sanger sequencing data, verification was performed using re-sequencing; PCR primer design and sequencing were performed as described above. One additional gap was present in a sequence representing a multicopy gene (i.e., a 23S rRNA sequence); no validation of this gap was attempted.

Verification of the 59 gaps by comparison with pre-existing trace files available in NCBI or by re-sequencing showed that all of these gaps represented alignment artifacts or artifacts due to the sequencing by synthesis technology. Thus, none of the short indels that were initially identified as differentiating the two 1998 from the two 2000 isolates or the two human from the two food isolates could be confirmed and these initially identified indels appear to represent sequencing errors, consistent with previous reports that the sequencing by synthesis strategy often incorrectly characterizes homopolymeric tracts [3].

### Validation of selected polymorphisms and plasmid presence using additional human isolates linked to the 2000 listeriosis outbreak

In order to identify additional human isolates linked to the 2000 outbreak, the Pathogen Tracker [80] database was used to identify human isolates that shared the same ribotype as the outbreak strain (DUP-1053A) and were isolated during the time frame of the outbreak (May to November of 2000) in some of the states (New York, Michigan and Ohio) that were included in the outbreak. Selected isolates identified through this approach were tested for rhamnose fermentation as well as by PFGE to identify specific isolates that matched the outbreak strain. Rhamnose fermentation was tested as previously described [81] with the exception that only one tube without a mineral oil overlay was used. PFGE with the restriction enzymes ApaI and AscI was performed using the US CDC standard PulseNet protocol [68]. Nine isolates matching the rhamnose fermentation characteristics and PFGE profile of the 1988 and 2000 isolates were selected to validate the polymorphisms described in Table 3 using PCR amplification and subsequent sequencing (see Additional file 3 for primers) of the regions containing these polymorphisms. All 9 of these isolates were also screened for the presence of two plasmid-specific sequences using PCR (see Additional file 3 for primers).

Unfortunately neither additional food nor additional human isolates linked to the 1988 case were available for analyses. Furthermore, no additional food or plant environmental isolates from 2000 or the period between 1998 and 2000 were available for analyses.

### Intracellular growth assay in activated J774 macrophage-like cells

*L. monocytogenes *strains were grown in BHI at 37°C with shaking at 220 RPM. Log phase (OD_600 _= 0.4) *L. monocytogenes *strains in BHI were diluted 1:100 and grown at 37°C to early stationary phase (defined as 3 h after the OD_600 _reached 1.0). Aliquots of the stationary phase cultures were flash frozen in liquid nitrogen and cultures were enumerated after thawing. The murine macrophage cell line J774A.1 (ATCC TIB-67) was maintained using Dulbecco's minimal essential medium (DMEM) with Earle's salts and 1% sodium pyruvate (Gibco; Gaithersburg, MD) containing 10% fetal bovine serum (Gibco), 1.5 g/L sodium bicarbonate (Gibco), and 100 μg/ml each penicillin G and streptomycin (J774 medium).

Intracellular growth assays were performed as described by Conte *et al*., [82] with minor modifications. Briefly, 48 h prior to the assay, J774 cells were seeded into 24 well plates (Corning) at a density of 2 × 10^5 ^cells/well in J774 medium without antibiotics. Twenty-four hours after seeding, the medium was replaced with J774 medium supplemented with 0.1 μg/ml *E. coli *O55:B5 LPS (Sigma). Thirty minutes prior to infection, the medium was replaced with fresh J774 medium without antibiotics. For infection, approximately 1 × 10^6 ^CFU *L. monocytogenes *were added to each well, resulting in an MOI of approximately 1. At 30 minutes post infection, the monolayers were washed once with PBS to remove any unassociated *L. monocytogenes*, and the medium was replaced with J774 media containing 50 μg/ml gentamycin to kill any extracellular *L. monocytogenes*. Infected J774 cells in different wells were washed 3 times with PBS and lysed with ice-cold distilled water after at 1, 5, 7, or 9 h post infection. Intracellular *L. monocytogenes *were enumerated by plating the appropriate dilutions of the J774 lysate on BHI.

Intracellular growth assays were initially performed separately with the two isolates to be tested (i.e., J0161 and J2818), but were also performed in a competition experiment format [36,37], where activated J774 cells were infected with both *L. monocytogenes *isolates (mixed at a 1:1 ratio). To assess the frequency of each strain after intracellular growth in the competition experiment, ten colonies recovered after 9 h of intracellular growth were selected from each of the three replicates for PCR amplification and sequencing of the region containing SNP 10 (Table 3) using primers RHO105-2327842F and RHO106-2327842R (Additional file 3).

## Authors' contributions

RHO perceived the study, performed genome alignments and genome alignment analyses, and re-sequencing of selected polymorphic sites; he drafted the manuscript. MLB formed and led the genome sequencing collaboration, participated in design and coordination of the study and helped draft the manuscript. SKY performed analysis and assembly of the 454 sequence data to create the genome sequences. CN led the 454 sequencing effort. JEG participated in the design and coordination of the study. BWB participated in the design and coordination of the study. PL developed and utilized the methods to rapidly isolate high quality genomic DNA from *L. monocytogenes *isolates and helped draft the manuscript. QS developed perl scripts required for analyses. RAI performed tissue culture experiments. LMG curated and provided bacterial isolates and researched isolate history. BS participated in the design and coordination of the study and helped draft the manuscript. MW participated in the design and coordination of the study and helped draft the manuscript. All authors read and approved the final manuscript.

## Supplementary Material

Additional file 1 **Intracellular growth of isolates J2818 and J0161 in activated J774 macrophage cells.** Graph of intracellular growth in activated macrophages of isolates J2818 and J0161. Click here for file

Additional file 2 **Comparison of *comK *prophage genes present in the human clinical and food isolates associated with the 1988 sporadic listeriosis case and the 2000 listeriosis outbreak.** This table provides a gene-by-gene analysis of the *comK *prophage in strain F6854 (representing the 1988 isolates), J2818 (representing the 2000 isolates), and other *L. monocytogenes *strains. Click here for file

Additional file 3 **Primers used for validation of polymorphisms.** List of primers used for validation of polymorphisms identified during genome analysis. Click here for file

Additional file 4 **Validation of the 43 polymorphic sites in the backbone alignment and the single polymorphic site in the tRNA prophage that were initially identified in the genome comparisons.** This table describes the 44 polymorphisms identified in the genome analysis, including whether a given polymorphism was confirmed by PCR amplification and subsequent sequencing of the PCR product. Click here for file
