# Supplementary material for: Short-term genome evolution of Listeria monocytogenes in a non-controlled environment
Source: BMC Genomics. 2008 Nov 13;9:539. doi: 10.1186/1471-2164-9-539 (PMC2642827; doi:10.1186/1471-2164-9-539)
Supplement: Additional file 1 — Intracellular growth of isolates J2818 and J0161 in activated J774 macrophage cells. Graph of intracellular growth in activated macrophages of isolates J2818 and J0161. [file 1471-2164-9-539-S1.doc]

**Additional file** **1. Intracellular growth of isolates J2818 and J0161 in activated J774 macrophage cells.** Two replicates were carried out for intracellular growth with each isolate (J0161 and J2818) individually (both replicates are shown in this graph). Three replicates were carried out for competitive assays using a mixture (1:1) of the two isolates and the average (and standard deviation) of the three replicates are shown. For each replicate of the competitive assay, 10 colonies were selected from BHI plates corresponding to T = 9 hours and a fragment corresponding to the *addB* SNP found in J2818 was PCR amplified and sequenced to assess the genotype of the colonies. Overall, 16 and 14 colonies, respectively, with the J2818 and the J0161 *addB* allelic type were recovered (3/10, 7/10, and 6/10 isolates carried the J2818 *addB* allelic type in replicates 1, 2, and 3, respectively). These data suggest that the mutations that differentiate the two 2000 isolates do not affect their intracellular growth capabilities as determined in activated J774 macrophage cells.
