# Supplementary material for: Short-term genome evolution of Listeria monocytogenes in a non-controlled environment
Source: BMC Genomics. 2008 Nov 13;9:539. doi: 10.1186/1471-2164-9-539 (PMC2642827; doi:10.1186/1471-2164-9-539)
Supplement: Additional file 2 — Comparison of comK prophage genes present in the human clinical and food isolates associated with the 1988 sporadic listeriosis case and the 2000 listeriosis outbreak. This table provides a gene-by-gene analysis of the comK prophage in strain F6854 (representing the 1988 isolates), J2818 (representing the 2000 isolates), and other L. monocytogenes strains. [file 1471-2164-9-539-S2.doc]

Additional file 2. Comparison of *comK* prophage genes present in the human clinical and food isolates associated with the 1988 sporadic listeriosis case and the 2000 listeriosis outbreak.

| **LMOf6854 Gene ID (annotation, nt length) for phage genes present in both 1988 isolates (i.e., isolates F6854 and F6900)^(1)^** | **J2818 Gene ID (nt length) for phage genes present in 2000 isolates^(2)^** | **% nt identity between genes in 1988 and 2000 isolates^(3)^** | ***L. monocytogenes* strain or prophage genes (except for F6854 and F6900) that showed highest similarity to the corresponding gene in 2000 isolates (% nt identity)^(4)^** |
| --- | --- | --- | --- |
| *Gene present, but not annotated in F6854* | 02217.2 (198 bp) | 100 | J1-194; EGD (100) |
| 2333.2 (gp30; 277 bp) | *Gene present, but not annotated in J2818* | 100 | J2-064 (100) |
| *Gene present, but not annotated in F6854* | 02218.2 (234 bp) | 100 | J1-194 (100) |
| 2333.3 (gp29; 372 bp) | 02219.2 (372 bp) | 100 | J2-003 (100) |
| 2333.4 (gp28; 450 bp) | 02220.2 (450 bp) | 100 | J2-064 (100) |
| 2338 (gp27; 498 bp) | 02221.2 (498 bp) | 100 | A006; R2-503 (97) |
| 2338.1 (gp26; 774 bp) | 02222.2 (774 bp) | 100 | A006 (100) |
| 2338.2 (L-alanoyl-D-glutamate peptidase; 846 bp) | 02223.2 (846 bp) | 94 | J1-194; A118 (95) |
| 2341 (holin; 282 bp) | 02224.2 (282 bp) | 99 | A118; J2-003 (98) |
| 2341.1 (gp23; 366 bp) | 02225.2 (366 bp) | 98 | J1-194; A118 (99) |
| 2341.2 (gp22; 159 bp) | 02226.2 (159 bp) | 98 | EGD-e (100) |
| 2341.3 (gp21; 318 bp) | 02227.2 (318 bp) | 92 | EGD-e; J1-194 (100) |
| 2341.4 (gp20; 1074 bp) | 02228.2 (1074 bp) | 97 (1029 bp) | 10403S (99) |
| 2341.5 (gp19; 1029 bp) | 02229.2 (1029 bp) | 97 | J1-194 (99) |
| 2341.6 (gp18; 1026 bp) | 02230.2 (1026 bp) | 100 | J2-003 (100) |
| 2341.7 (gp17; 819 bp) | 02231.2 (801 bp) | 97 | J1-194 (100)^R1^ |
| 2341.8 (tape-measure; 5364 bp) | 02232.2 (5364 bp) | 94 (4322 bp) | J1-194 (98)^R1^ |
| 2341.9 (gp15; 606 bp) | 02233.2 (606 bp) | 100 | EGD-e; A118; J2-003 (99) |
| 2341.10 (gp14; 423 bp) | 02234.2 (423 bp) | 100 | CLIP11262; A500 (97) |
| 2352 (major tail protein; 294 bp) | 02235.2 (333 bp) | 100 | CLIP11262 (91) |
| 2352.1 (major tail shaft protein 438 bp) | 02236.2 (435 bp) | 93 (432 bp) | A118; LO28 (93) |
| 2352.2 (gp11; 408 bp) | 02237.2 (408 bp) | 88 | J1-194 (100)^R2^ |
| 2352.3 (gp10; 339 bp) | 02238.2 (339 bp) | 86 (210 bp) | J1-194 (100)^R2^ |
| 2352.4 (gp9; 363 bp) | 02239.2 (363 bp) | 91 (324 bp) | J1-194 (100)^R2^ |
| 2352.5 (gp8; 396 bp) | 02240.2 (396 bp) | 88 (392 bp) | J1-194 (100)^R2^ |
| 2352.6 (gp7; 159 bp) | - | NC | NC^R2^ |
| 2352.7 (Major head protein; 837 bp) | 02241.2 (1002 bp) | NH | J1-194 (99)^R2^ |
| 2360 (scaffolding protein; 600 bp) | 02242.2 (591 bp) | 87 (143 bp) | J1-194 (100)^R2^ |
| 2360.1 (gp4; 1140 bp) | 02243.2 (1128 bp) | 90 (1119 bp) | J1-194 (96)^R2^ |
| 2360.2 (phage portal protein; 1446 bp) | 02244.2 (1761 bp) | 94 (1406 bp) | LO28 (92) |
| 2363 (phage terminase; 1311 bp) | 02245.2 (1311 bp) | 98 | N1-017 (96) |
| 2364 (terminase small subunit; 714 bp) | 02246.2 (756 bp) | 99 | N1-017 (99) |
| 2364.1 (gp68; 711 bp) | 02247.2 (540 bp) | NH | EGD-e (100) |
| 2366 (putative positive control factor; 573 bp) | 02248.2 (435 bp) | NH | HPB2262 (98) |
| 2366.1 (lin2398-related; 156 bp) | 02249.2 (165 bp) | NH | A500 (95) |
| 2366.2 (c.h.p.; 414 bp) | 02250.2 (405 bp) | NH | A006; A118 (98) |
| 2366.3 (similar to phage protein; 276 bp) | 02251.2 (321 bp) | NH | A006; A118 (97) |
| 2366.4 (c.h.p.; 192 bp)* | 02252.2 (384 bp) | NH | NH |
| 2371 (single-strand binding protein subfamily (ssb); 483 bp) | 02253.2 (483 bp) | 92 | EGD-e; J2-003 (92) |
| 2371.1 (c.h.p.; 201 bp) | 02254.2 (180 bp) | 100 | EGD-e; J2-003 (100) |
| 2371.2 (c.h.p.; 168 bp) | - | NC | NC |
| 2371.3 (c.h.p.; 462 bp) | - | NC | NC |
| 2371.4 (c.h.p.; 471 bp) | 02255.2 (471 bp) | 100 | EGD-e; J2-003 (100) |
| 2371.5 (c.h.p.; 444 bp) | 02256.2 (444 bp) | 100 | EGD-e (100) |
| 2371.6 (c.h.p.; 198 bp) | 02257.2 (198 bp) | 100 | EGD-e; J2-003 (100) |
| 2371.7 (gp51; 597 bp) | 02258.2 (597 bp) | 99 | EGD-e (100) |
| 2379 (DNA methyltransferase superfamily; 813 bp) | 02259.2 (984 bp) | NH | HPB2262 (90) |
| *Gene absent in F6854* | 02260.2 (255 bp) | NC | PSA (97) |
| 2380 (DnaD domain protein; 945 bp) | 02261.2 (930 bp) | NH | J1-194 (99; 806 bp)^R3^ |
| 2381 (recombinase; 816 bp) | 02262.2 (810 bp) | NH | J1-194 (100)^R3^ |
| 2381.1 (gp47; 960 bp) | 02263.2 (882 bp) | NH | J1-194 (100)^R3^ |
| 2381.2 (c.h.p.; 189 bp) | *Gene present, but not annotated in J2818* | 85 (120 bp) | J1-194 (100)^R3^ |
| *Gene absent in F6854* | 02264.2 (204 bp) | NC | J1-194 (100)^R3^ |
| 2381.3 (DNA metabolism; 225 bp)* | 02265.2 (216 bp) | 94 | J1-194 (100)^R3^ |
| 2381.4 (gp43; 534 bp)* | 02266.2 (534 bp) | 87 | J1-194 (100)^R3^ |
| 2385 (Phage antirepressor protein subfamily 777 bp) | 2267.2 (780 bp) | 89 (552 bp) | J1-194 (100)^R3^ |
| 2385.1 (c.h.p.; 285 bp) | 02268.2 (252 bp) | NH | J1-194; R2-503; HPB2262 (100)^R3^ |
| 2385.2 (gp41; 282 bp) | *Gene present, but not annotated in J2818* | 94 (206 bp) | J1-194; R2-503 (100)^R3^ |
| *Gene absent in F6854* | 02269.2 (162 bp) | NC | J1-194; R2-503 LO28; F2-515; CLIP11262 (100)^R3^ |
| 2385.3 (c.h.p.; 285 bp) | LMOG_02949.1 (231 bp) | 87 (124 bp) | J1-194; A118 (100)^R3^ |
| *Gene absent in F6854* | LMOG_02948.1 (267 bp) | NC | J1-194; HPB2262; F2-515; A118 (100)^R3^ |
| 2387 (transcriptional regulator, Cro-CI family; 243 bp) | LMOG_02947.1 (279 bp) | NH | J1-194; A118 (100)^R3^ |
| 2388 (transcriptional regulator, Cro-CI family; 459 bp) | 02832.2 (306 bp) | NH | J1-194; HPB2262; A118 (100)^R3^ |
| *Gene absent in F6854* | 02833.2 (492 bp) | NC | J1-194; HPB2262; A118 (100)^R3^ |
| *Gene absent in F6854* | 0.2834.2 (218 bp) | NC | J1-194; HPB2262; A118 (100)^R3^ |
| 2388.1 (gp33; 702 bp) | 02835.2 (723 bp) | 86 (318 bp) | J1-194; HPB2262 (100)^R3^ |
| 2388.2 (gp32; 681 bp) | 02836.2 (741 bp) | NH | J1-194; A118 (100)^R3^ |
| 2391 (prophage LambdaW5, site-specific recombinase, resolvase family; 1359 bp) | 02837.2 (1359 bp) | 98 | J1-194 (100)^R3^ |

^(1)^ h.p., hypothetical protein; c.h.p., conserved hypothetical protein; the two genes marked with a * were not identified in the genome sequence for F6900 (1988 human isolate); the genes upstream and downstream of these genes fall into different contigs strongly suggesting that these genes were not sequenced in the genome draft for F6900 used for this study. “*Gene absent on F6854*” indicates that the sequence of a gene present in the 2000 isolates is not present in F6854 (1988 food isolate) and there in no other gene in F6854 in the respective region.

^(2)^gene IDs for the 2000 food isolate J2818 (i.e., LMPG numbers as assigned by the Broad Institute) are shown with gene length in parenthesis. Genes that were not sequenced or annotated in J2818 but were present in J0161 (2000 human isolate) are shown with their respective J0161 ID (e.g. LMOG_02947.1). “-“ indicates that the gene is missing in the 2000 isolates. “*Gene present but not annotated in J2818*” indicates that the sequence of a gene annotated in F6854 (1988 food isolate) is present in the 2000 isolates but was not annotated as a gene.

^(3)^ % nt identity is given; for homologies calculated for less than to full sequence length, the length of the fragment for which nt identity was calculated is shown in parenthesis; NH, no homology was observed between F6854 and J2818 or J0161 but a gene is present in the respective region; NC, not calculated (for genes that were absent in the 2000 isolates or in F6854); All identities were calculated using the genes in the *comK* prophage of J2818 and J0161 as queries in blastn searches against the genomes of F6854

^(4)^ The *L. monocytogenes* strain or bacteriophage that showed the highest nt similarity with the prophage gene in the 2000 isolate is shown (matches to the corresponding gene in one of the 1998 isolates, i.e., F6854 or F6900, are not shown). A118, A500, A006, and PSA are listeriaphages, while EGD-e, HPB2262, LO28, FSL J2-006, FSL N1-017, FSL R2-503, FSL J1-194 are *L. monocytogenes* strains (FSL prefixes were omitted in the table); CLIP11626 is a *L. innocua* strains. For homologies calculated for less than to full sequence length, the length of the fragment for which nt was calculated is shown in parenthesis; NH, no homology; NC, not calculated (for genes that were absent in the 2000 isolates). R1, R2, and R3 superscripts indicate genes located in the three recombinant fragments depicted in Figure 2.
