# Supplementary material for: Short-term genome evolution of Listeria monocytogenes in a non-controlled environment
Source: BMC Genomics. 2008 Nov 13;9:539. doi: 10.1186/1471-2164-9-539 (PMC2642827; doi:10.1186/1471-2164-9-539)
Supplement: Additional file 3 — Primers used for validation of polymorphisms. List of primers used for validation of polymorphisms identified during genome analysis. [file 1471-2164-9-539-S3.doc]

Additional file 3. Primers used for validation of polymorphisms

| Pair* | Primer name (SNP confirmed, as in Table 3) | Sequence (5'-3') | Annealing T (ºC) |
| --- | --- | --- | --- |
| 1-P | RHO41-174721F (4) | ACTTATTTAGCGCTTTTCCCAGG | 55.1 |
| 1-P | RHO42-174721R (4) | ACGGCATCTACCAAATCAAACAG | 54.9 |
| 2-P | RHO43-279603F | GGAGATGTAGCAGAGAAAACGGG | 55.9 |
| 2-P | RHO44-279603R | TGCATGAATCCTTTAAAACGGTAATC | 56.7 |
| 3-P | RHO45-378489F (2) | AAAAACTGGCGATAATGGAGATAGTC | 55.4 |
| 3-P | RHO46-378489R (2) | CTTCATAACGAGTGGGAGTAATGTAGAC | 55.1 |
| 4-P | RHO47-392427F (5) | CTTTTAACTGCGGTAGGGAATGTG | 56 |
| 4-P | RHO48-392427R (5) | CAAAAGGCGCTAAATAACCAAGTAAC | 56.1 |
| 5-P | RHO49-462089F (6) | GTCAGCGGAAACTAAACCAGAAAC | 55.3 |
| 5-P | RHO50-462089R (6) | CGAACCTTAGCGGAAAACAATG | 55.5 |
| 6-P | RHO51-597473F | CCCCTTGACCCCGTTGTAG | 54.1 |
| 6-P | RHO52-597473R | TCGGCGCCATTTTTCATAAC | 55.1 |
| 7-P | RHO53-885898F (7) | CATGGCGGCCCTCTTTTC | 55.3 |
| 7-P | RHO54-885898R (7) | CCCGCGATTCCCATACAAC | 55 |
| 8-P | RHO55-910350F | CCGCACTTGACCCTGAAATG | 55.3 |
| 8-P | RHO56-910350R | CGTCGGCGGATCTGTGAAG | 56.3 |
| 9-P | RHO57-991247F | GACTTCTTCGGCGTTAAAAATGTG | 56.1 |
| 9-P | RHO58-991247R | AAACGAGTAATGGAATGGATTGATAGAC | 56.4 |
| 10-P | RHO59-1079983F | AGGCGGAAGGTATTAAGCAGATG | 55.7 |
| 10-P | RHO60-1079983R | GTCTATCGATCAAAGTACCTGGACAAG | 55.7 |
| 11-P | RHO61-1172396F | GTGCACTAGATGCGGTTGAGATAG | 54.8 |
| 11-P | RHO62-1172396R | CTCTACTGGCTTCATAGGATTTACTGG | 55.3 |
| 12-P | RHO63-1193390F (8) | GACGGCGCATCTTTTGACC | 55.4 |
| 12-P | RHO64-1193390R (8) | AGGCGTTGCAATTGTGAAAGTAG | 55.2 |
| 13-P | RHO65-1573462F | CCAATCCCCGCAATTTCTTTAG | 56.6 |
| 13-P | RHO66-1573462R | ATGGGCGGTGCATTAGGTG | 55.5 |
| 14-P | RHO67-1575291F | CGAACCGCCACCCTTTTTG | 57.6 |
| 14-P | RHO68-1575291R | CCCCTGTGATGGCGAGTCTTAG | 57.4 |
| 15-P | RHO69-1625104F (9) | ATCCCCATCCCAGAAACACG | 55.8 |
| 15-P | RHO70-1625104R (9) | GGCCGCCAAGCAAAAGAG | 55.7 |
| 16-P | RHO71-1675779F (1) | CTGTAAAAACAGTTCACTTAATCCATCC | 55.1 |
| 16-P | RHO72-1675779R (1) | AGCTTGTTGCTTTGCCGTATTC | 55.3 |
| 17-P | RHO73-1888180F | GGTTCGCGAGTTTTATCATCTTTG | 56 |
| 17-P | RHO74-1888180R | TTTTTTTGATTGACAAGGAGAGTTCTATC | 56.1 |
| 18-P | RHO75-1896904F | CAGTTCAATAATTCGGCGGTCTAC | 55.9 |
| 18-P | RHO76-1896904R | AGGAAATCCTGGTGGGTTACTTG | 55.2 |
| 19-P | RHO77-2053116F (3) | AAAAGCGTCGTTTGTTTGGAATAG | 55.8 |
| 19-P | RHO78-2053116R (3) | TCATCGCTGGAAAATCTGGTTATAC | 55.5 |
| 20-P | RHO79-2304425F | TTATCTTGCGGCTGTTGATGG | 55.2 |
| 20-P | RHO80-2304425R | TGGCGGTAAAAATCATTATGCTG | 55.8 |
| 21-P | RHO81-2372845F (11) | TATGTTTGGCGTGAAAGAAGAGC | 55.3 |
| 21-P | RHO82-2372845R (11) | ATCGAAGGCATGGACGTAGTAGAC | 55.5 |
| 22-P | RHO83-2389379F | CCACCGGAAAAAGCGACTAAG | 55.5 |
| 22-P | RHO84-2389379R | ATGCATATTTGGGCCGTAACTC | 54.7 |
| 23-P | RHO85-2391444F | AGATGAAAATGGCGATGAATTAGC | 55.6 |
| 23-P | RHO86-2391444R | CGGCGGAAGTTTGATAAGGTG | 56.1 |
| 24-P | RHO87-2454994F | CTAAAACCGCCCAATTTGACTAAG | 55.4 |
| 24-P | RHO88-2454994R | TTTTGAGCGAATGAAACGACAAC | 55.9 |
| 25-P | RHO89-2566002F | AGCCGAGTCAAAACAGCAACAG | 56 |
| 25-P | RHO90-2566002R | CAGCGCGTATTAAGCAAGGAAC | 55.8 |
| 26-P | RHO91-2590249F | GCTTTATTGGATGAAATGGCTGC | 56.7 |
| 26-P | RHO92-2590249R | TGGCGCGAAAAAGAACTAACG | 56.9 |
| 27-P | RHO93-2601824F | AACCGACGACCCCTTCCTTAC | 55.9 |
| 27-P | RHO94-2601824R | CCGGCGAGTTACGATTTGTTG | 56.9 |
| 28-P | RHO95-2610499F | ATTGCGGCTTGGTTATTAGGTTC | 55.5 |
| 28-P | RHO96-2610499R | TGTTGGCGGGGTAGGGAG | 55.5 |
| 29-P | RHO97-2612921F | TCCGCCTCCTTCTCCCTTC | 55.5 |
| 29-P | RHO98-2612921R | TGCCGCTACAACATCATCCAC | 55.8 |
| 30-P | RHO99-2649889F | GATCGCCATTGTTTCCCCTC | 55.6 |
| 30-P | RHO100-2649889R | CCCTATGGCCCACGGTTC | 54.8 |
| 31-P | RHO101-2654727F | AATTAGGGCCAGCCGTTTTG | 55.6 |
| 31-P | RHO102-2654727R | ATCATTAGGTTCTCCGCCCATAC | 55.2 |
| 32-P | RHO103-5362_PHAGE2F (12) | GTCCAATCTGCCGCTTCAAAC | 56.1 |
| 32-P | RHO104-5362_PHAGE2R (12) | TGAGGCAGGGAAAGTTACTAAAACATAC | 56.7 |
| 33-P | RHO105-2327842F (10) | TCCATCCCTGTTTTTGCCTTC | 55.5 |
| 33-P | RHO106-2327842R (10) | GATACCGCGACATAGCTATTTTGAC | 55.3 |
| 34-P | RHO107-2391444_newF | CAAACAGTCACTTACATTTATGAAAAAGC | 55.4 |
| 34-P | RHO108-2391444_newR | CGGCGGAAGTTTGATAAGGTG | 56.1 |
| 35-G | RHO109-888300F | TTGTATCGCATGTGCTTTTACCAG | 56 |
| 35-G | RHO110-888300R | AATAAGTCTTTGCCTGTTTCATCTACG | 55.7 |
| 36-G | RHO111-959341F | AGCATCGCGATCAAGATTTCC | 55.7 |
| 36-G | RHO112-959341R | TTTATTACGGAGAAGGAGGGATTTG | 56 |
| 37-G | RHO113-1033180F | CGCTTTTGATTGGTTTTCGTTC | 55.5 |
| 37-G | RHO114-1033180R | CGGACTTCACATGGTTTTCTGG | 55.9 |
| 38-G | RHO115-1080842F | GGCTGAGATATGCGGATGGTG | 56.7 |
| 38-G | RHO116-1080842R | TGGAAAAGCTGGCGTAAGTGAAG | 57.4 |
| 39-G | RHO117-1360181F | CATGAACGCGTTGGGAAAGTAG | 56.2 |
| 39-G | RHO118-1360181R | GTCCAAGCGGCATTAGAAGATG | 55.4 |
| 40-G | RHO119-1895767F | GCACCGCAAGCACATTTCC | 56.3 |
| 40-G | RHO120-1895767R | ACGCGCCGGTAGCATTTG | 56.9 |
| 41-R | RHO121-phage1F^†^ | TGAGGCAGGGAAAGTTACTAAAACATAC | 57.6 |
| 41-R | RHO122-phage1R^†^ | GCGAACACGAACAGTCTAGGATTAGTAG | 57.2 |
| 42-L | RHO123-Plasmid2F | AAAGAATACGTCGTGCTGCTG | 52.3 |
| 42-L | RHO124-Plasmid2R | TTGAAATTGTTGGTTCTTATACTGAAG | 52.5 |
| 43-L | RHO125-Plasmid1F | TTATTATGTGTTCATTCGCAAGC | 51.7 |
| 44-L | RHO-126-Plasmid1R | ATCCACGTTTAACGCACTCC | 51.7 |

* Primers used to validate point substitutions (P), gaps (G) or recombination (R).
