# Supplementary material for: Short-term genome evolution of Listeria monocytogenes in a non-controlled environment
Source: BMC Genomics. 2008 Nov 13;9:539. doi: 10.1186/1471-2164-9-539 (PMC2642827; doi:10.1186/1471-2164-9-539)
Supplement: Additional file 4 — Validation of the 43 polymorphic sites in the backbone alignment and the single polymorphic site in the tRNA prophage that were initially identified in the genome comparisons. This table describes the 44 polymorphisms identified in the genome analysis, including whether a given polymorphism was confirmed by PCR amplification and subsequent sequencing of the PCR product. [file 1471-2164-9-539-S4.doc]

Additional file 4. Validation of the 43 polymorphic sites in the backbone alignment and the single polymorphic site in the tRNA prophage that were initially identified in the genome comparisons

| **Poly-morphism no.^(1)^** | **Position of poly-morphism ^(2)^** | **Con-firmation (SNP no.)** | **Summary of result^(3)^** |
| --- | --- | --- | --- |
| I | 174721 | Confirmed (SNP #4) | Confirmed as T→C in J2818. Validated by re-sequencing and Sanger |
| II | 279603 | Not confirmed | Mismatch due to 454 sequencing error in F6900. Miscalling of the number of bases in homopolymeric tract. Verified by re-sequencing |
| III | 378489 | Confirmed (SNP #2) | Confirmed as C→T in J0161. Validated by re-sequencing and Sanger |
| IV | 392427 | Confirmed (SNP #5) | Confirmed as G→A in J2818. Validated by re-sequencing |
| V | 462089 | Confirmed (SNP #6) | Confirmed as G→A in J2818. Validated by re-sequencing |
| VI | 597473 | Not confirmed | Mismatch due to 454 sequencing error. Miscalling of the correct nucleotide in J2818. Verified by re-sequencing and Sanger |
| VII | 885898 | Confirmed (SNP #7) | Confirmed as A→G in J2818. Validated by re-sequencing |
| VIII | 910350 | Not confirmed | Mismatch due to 454 sequencing error in F6900. Miscalling of the number of bases in homopolymeric tract. Verified by re-sequencing |
| IX | 910351 | Not confirmed | Mismatch due to 454 sequencing error in F6900. Carry forward of nucleotide in homopolymeric tract. Verified by re-sequencing |
| X | 910382 | Not confirmed | Mismatch due to 454 sequencing error in J0161. Miscalling of the number of bases in homopolymeric tract. Verified by re-sequencing and Sanger |
| XI | 991247 | Not confirmed | Mismatch due to 454 sequencing error in F6900. Carry forward of nucleotide in homopolymeric tract. Verified by re-sequencing |
| XII | 991248 | Not confirmed | Mismatch due to 454 sequencing error in F6900. Carry forward of nucleotide in homopolymeric tract. Verified by re-sequencing |
| XIII | 1079983 | Not confirmed | Misalignment of partially sequenced region in F6900 |
| XIV | 1172396 | Not confirmed | Misalignment of repetitive region partially sequenced in F6900. |
| XV | 1193390 | Confirmed (SNP #8) | Confirmed as C→A in J2818. Validated by re-sequencing |
| XVI | 1573462 | Not confirmed | Miscalling of the right nucleotide in F6854. Verified by re-sequencing and Sanger |
| XVII | 1575291 | Not confirmed | Miscalling of the right nucleotide in F6854. Verified by re-sequencing and Sanger |
| XVIII | 1625104 | Confirmed (SNP #9) | Confirmed as G→T in J2818. Validated by re-sequencing |
| XIX | 1675779 | Confirmed (SNP #1) | Confirmed as A→G in F6900; Validated by re-sequencing |
| XX | 1888180 | Not confirmed | Miscalling of the right nucleotide in F6854. Verified by re-sequencing and Sanger |
| XXI | 1896904 | Not confirmed | Miscalling of the right nucleotide in F6854. Verified by re-sequencing and Sanger |
| XXII | 2053116 | Not confirmed in FSL R2-499^(4)^ but confirmed in J0161 (SNP #3) | Not confirmed by re-sequencing of FSL R2-499 (a clone of J0161) but confirmed when verified by Sanger. T→C in J0161 |
| XXIII | 2304425 | Not confirmed | Mismatch due to 454 sequencing error in F6900. Carry forward of nucleotide in homopolymeric tract. Verified by re-sequencing |
| XXIV | 2304426 | Not confirmed | Mismatch due to 454 sequencing error in F6900. Carry forward of nucleotide in homopolymeric tract. Verified by re-sequencing |
| XXV | 2327842 | Confirmed (SNP #10) | Confirmed as C→A in J2818. Validated by re-sequencing |
| XXVI | 2401731 | Confirmed (SNP #11) | Confirmed as G→A in J2818. Validated by re-sequencing |
| XXVII | 2418265 | Not confirmed | Misalignment of repetitive region partially sequenced in F6900. Verified by re-sequencing |
| XXVIII | 2420330 | Not confirmed | Misalignment of repetitive region partially sequenced in J2818. Verified by re-sequencing |
| XXIX | 2420336 | Not confirmed | Misalignment of repetitive region partially sequenced in J2818. Verified by re-sequencing |
| XXX | 2420339 | Not confirmed | Misalignment of repetitive region partially sequenced in J2818. Verified by re-sequencing |
| XXXI | 2420351 | Not confirmed | Misalignment of repetitive region partially sequenced in J2818. Verified by re-sequencing |
| XXXII | 2420367 | Not confirmed | Misalignment of repetitive region partially sequenced in J2818. Verified by re-sequencing |
| XXXIII | 2447535 | Not confirmed | Misalignment of partially sequenced region in J0161. Verified by Sanger |
| XXXIV | 2483880 | Not confirmed | Miscalling of the right nucleotide in F6854. Verified by re-sequencing |
| XXXV | 2501317 | Not confirmed | Mismatch due to 454 sequencing error in J2818. Miscalling of the number of bases in homopolymeric tract. Verified by Sanger |
| XXXVI | 2594888 | Not confirmed | Misalignment of partially sequenced region. Verified by re-sequencing |
| XXXVII | 2619135 | Not confirmed | Miscalling of the right nucleotide in F6854. Verified by re-sequencing |
| XXXVIII | 2635720 | Confirmed (SNP #12) | Confirmed as A→T in J2818 and J0161. Validated by re-sequencing |
| XXXIX | 2665413 | Not confirmed | Misalignment of repetitive sequence in F6854. Verified by Sanger |
| XXXX | 2674088 | Not confirmed | Mismatch due to 454 sequencing error in F6900. Miscalling of the number of bases in homopolymeric tract. Verified by re-sequencing |
| XXXXI | 2674089 | Not confirmed | Mismatch due to 454 sequencing error in F6900. Miscalling of the number of bases in homopolymeric tract. Verified by re-sequencing |
| XXXXII | 2676510 | Not confirmed | Mismatch due to 454 sequencing error in J2818. Miscalling of the number of bases in homopolymeric tract. Verified by re-sequencing |
| XXXXIII | 2713478 | Not confirmed | Miscalling of the right nucleotide in F6854. Verified by re-sequencing |
| XXXXIV | 2718316 | Not confirmed | Miscalling of the right nucleotide in F6854. Verified by re-sequencing |

^(1)^Initial polymorphisms identified based on the genome sequences were labeled with Roman numerals to avoid confusion with the confirmed SNPs (as shown in Table 3, which were labeled using Arabic numerals).

^(2)^nt position in the full alignment of F6854, F6900, J0161 and J2818;

^(3)^Sanger refers to Sanger trace files available on NCBI;

^(4)^FSL R2-499 is a subculture of J0161 sent from CDC to the Food Safety Laboratory at Cornell University;
